# Identification of a Putative Enhancer RNA for EGFR in Hyper-Accessible Regions in Esophageal Squamous Cell Carcinoma Cells by Analysis of Chromatin Accessibility Landscapes

**DOI:** 10.3389/fonc.2021.724687

**Published:** 2021-10-15

**Authors:** Sangyong Choi, Adwait Sathe, Ewy Mathé, Chao Xing, Zui Pan

**Affiliations:** ^1^College of Nursing and Health Innovation, University of Texas at Arlington, Arlington, TX, United States; ^2^Department of Nutritional Sciences, College of Agriculture, Health and Natural Resources, University of Connecticut, Storrs, CT, United States; ^3^Eugene McDermott Center for Human Growth and Development, University of Texas Southwestern Medical Center, Dallas, TX, United States; ^4^Department of Biomedical Informatics, The Ohio State University Wexner Medical Center, Columbus, OH, United States; ^5^Division of Preclinical Innovation, National Center for Advancing Translational Sciences, NIH, Rockville, MD, United States; ^6^Department of Population and Data Sciences, University of Texas Southwestern Medical Center, Dallas, TX, United States; ^7^Department of Bioinformatics, University of Texas Southwestern Medical Center, Dallas, TX, United States

**Keywords:** esophageal cancer, ATAC-seq, AP-1, digital droplet PCR, tyrosine kinase inhibitor (TKI), non-coding RNA (ncRNA), enhancer RNA (eRNA), transcription factor binding

## Abstract

Abnormal genetic and epigenetic modifications play a key role in esophageal cancer. By Assay for Transposase-Accessible Chromatin by sequencing (ATAC-seq), this study compared chromatin accessibility landscapes among two esophageal squamous cell carcinoma (ESCC) cell lines, KYSE-30 and KYSE-150, and a non-cancerous esophageal epithelial cell line, HET-1A. Data showed that hyper-accessible regions in ESCC cells contained genes related with cancer hallmarks, such as epidermal growth factor receptor (EGFR). Multi-omics analysis and digital-droplet PCR results demonstrated that several non-coding RNAs in EGFR upstream were upregulated in ESCC cells. Among them, one appeared to act as an enhancer RNA responsible for EGFR overexpression. Further motif analysis and pharmacological data suggested that AP-1 family transcription factors were able to bind the hyper-accessible regions and thus to regulate cancer cell proliferation and migration. This study discovered a putative enhancer RNA for EGFR gene and the reliance of ESCC on AP-1 transcription factor.

## 1 Introduction

Esophageal cancer is among the most fatal cancers and presents two main sub-types, i.e., esophageal adenocarcinoma (EAC) and esophageal squamous cell carcinoma (ESCC). The development of ESCC is strongly influenced by genetic and environmental factors *via* epigenetic changes. Such epigenetic changes, including alterations in transcriptome and DNA methylome, have been revealed by genome-wide RNA transcripts and CpG site methylation tests (1–3). Also, a large proportion of ESCC-associated SNPs located at regulatory regions other than exon have been found to control target gene transcription (4). Despite these findings, a panoramic view of deregulated genetic and epigenetic networks in ESCC cells is missing, which is essential to dissect the molecular mechanisms contributing to cancer development and progression. One way to provide such comprehensive information of genetic and epigenetic networks in cancer cells is through chromatin landscape, especially through open chromatin regions with high DNA accessibility. A map of open chromatin is able to uncover all classes of cis-regulatory elements, such as active promoters, enhancers, and insulators. Moreover, given that transcription factors bind to DNA in a sequence-specific manner by removing or moving the nucleosomes to form open chromatin, mapping hyper-accessible regions in cancer cell genome provides information regarding onco-transcription and pioneering factors (5).

Owing to the advances of next-generation sequencing (NGS), chromatin landscape can be established by a few high-throughput approaches, such as MNase-seq, DNase-seq, FAIRE-seq, and ATAC-seq. ATAC-seq, Assay for Transposase-Accessible Chromatin using sequencing, is a recently developed powerful tool with several advantages (6). It employs the enzyme Tn5 transposase to bind to and cleave accessible DNA and simultaneously tags oligo adaptors at the beginning or end of the chromatin fragments. The pool of DNA fragments of 60–120 bp long generated from the nucleosome-free regions (NFR) or of ~150-bp increments generated from the nucleosome-containing chromatin (NCC) is collected and followed by NGS. The first advantage of ATAC-seq is that it can simultaneously recover most chromatin structures, from NFR to NCC containing one, two, or multiple nucleosomes. Therefore, ATAC-seq is able to show the more complex chromatin structures, such as nucleosome positioning and packing, and transcription factor occupancy. The second advantage of ATAC-seq is that the sample library preparation is easy and rapid. The whole process can be completed within a few hours compared with 1–2 days for other -seq approaches, such as FAIRE-Seq or DNase-Seq. Thirdly, the construction of ATAC-seq libraries requires only a few thousand cells and even single-cell ATAC-seq is possible. As such, ATAC-seq becomes attractive for high-throughput analysis with rare clinical samples or biological specimens.

Among many oncogenes and oncogenic pathways, the epidermal growth factor receptor (EGFR), and its signaling network, is one of the most important ones. As well as many ESCC cell lines, over 70% of patient tissues exhibited the overexpression and amplification of EGFR, which is also strongly associated with aggressive cancer features and poor prognosis (7, 8). Moreover, targeting EGFR using specific tyrosine kinase inhibitors (TKIs) or monoclonal antibodies has been suggested as a neoadjuvant therapeutic intervention along with surgery, radiation, and/or chemotherapy. However, TKIs or EGFR antibodies, such as gefitinib and cetuximab, respectively, have exhibited limited or no clinical benefits for ESCC patients (9, 10), implying the necessities of further understanding EGFR regulation in ESCC cells in order to develop improved or new strategies targeting EGFR as therapeutic measures for ESCC patients.

This study aims to generate an integrated transcriptional and epigenetic regulatory network of ESCC using ATAC-seq. Results were refined with pertinent publicly available ChIP-seq, CAGE/GRO/PRO-seq, and the Cancer Genome Atlas (TCGA) expression data. We then employed bioinformatic analyses to study the chromatin dysregulation of EGFR locus with an emphasis on searching for novel non-coding RNA (ncRNA) functioning as an enhancer RNA. Lastly, *via* DNA motif analysis, we identified that AP-1 is associated with open chromatin in ESCC cells. Further studies using pharmacological AP-1 inhibitor confirmed its association on subsequent EGFR overexpression, cell proliferation, and migration in ESCC cells.

## 2 Materials and Methods

### 2.1 Cell Cultures

HET-1A cells were cultured in the base medium for this cell line (BEBM) with all the additives as American Type Culture Collection (ATCC) recommends. KYSE-30 and KYSE-150 cells were grown in RPMI/F12 + 5% FBS + 1% antibiotic–antimycotic (Thermo Fisher Scientific) (11).

### 2.2 ATAC-seq

ATAC-seq libraries were prepared following published protocol (6). Cells were harvested for ATAC-seq by centrifugation and re-suspended in cold PBS. Fifty thousand cells were immediately lysed using cold lysis buffer, and isolated nuclei from the cells were incubated in the Tn5-mediated transposase reaction mix for 30 min at 37°C. Following purification, the fragmented DNA library from each cell line was barcoded by PCR using customized Illumina sequencing primers. Library complexities were validated by Bioanalyzer, and libraries were quantified by qRT-PCR. The normalized amounts of libraries from all cell replicates were combined and submitted to the Next-generation Sequencing Core at the University of Texas at Southwestern. Sequencing experiment was performed using 75x75 High-Output kits on NextSeq 550 system. Normalized ATAC-seq data have been deposited in the GEO database (GSE162380).

### 2.3 Bioinformatics Analysis

The quality trimmed (ea-utils/1.1.2-806) (12), duplicate marked (picard-tools v2.2.1) reads were mapped to hg19 human genome (bowtie2 v 2.3.3.1) (13), and blacklist regions were filtered (bedtools v 2.7.1) and Tn5 shifted. In this analysis, sub-nucleosomal, i.e., <100-bp, reads were used and peaks were called using MACS 2.1.0 with the following parameters: *q*-value = 0.05, –nomodel – shift -100 – extsize 200 with BED formatted input. The ATAC-seq counts were first normalized using the DiffBind default DESeq2 normalization method. Differential binding analysis was performed on a merged consensus set of open chromatin regions (OCRs) in all the samples using DiffBind4 package (14, 15). This was carried out for each pair among the three conditions KYSE-30, KYSE-150, and HET-1A. ATAC-seq open chromatin regions are defined by using the annotation tool, ChIP seeker (16). The standard definition is −3k/+3k bp from an annotated TSS for a promoter region. *z*-scores are defined as: (ATAC-Seq region normalized counts in sample of interest) − (Mean normalized counts across all samples)/standard deviation. The *z*-scores were calculated on a per-OCR basis across samples and represented in a heatmap using base R (http://www.R-project.org). Hierarchical clustering was carried out using Pearson correlation as a distance measure. Information of all differential OCRs in each cluster such as gene coordinates, annotation, gene symbols, and distance to transcription start site are included in **Table S3**. *De novo* and known motif over-representation analysis in the selected merged OCR sets was done using the findMotifsGenome.pl script in the HOMER collection (17). Web-based functional enrichment analysis tool, WebGestalt was used for generating functional enrichment terms for nearest-neighbor genes for peaks (18). The top 20 most significant categories per Subcluster were used for the enrichment categories from the following databases: GO (Biological Process) (19, 20), KEGG (21), Reactome (22), Wikipathways (cancer) (23), and Disgenet (24). Data from RNA-seq datasets of esophageal carcinoma patients (ESCA) and normal esophageal tissues were extracted from “The Cancer Genome Atlas (TCGA)” and “The Genotype-Tissue Expression (GTEx)” database using GEPIA2 (http://gepia2.cancer-pku.cn) (25). Transcript expression levels for specific genes in both the tumor and neighboring tissues from esophageal cancer patients on the TCGA and normal esophageal tissues on the GTEx were pulled out and visualized as boxplots. ChIP-Atlas (https://chip-atlas.org) was utilized to obtain ChIP-seq datasets: various ESCC cells for histones and digestive tract cells (DLD-1, HCT116, HT-29, and SW480) for RNA polymerase 2 (POL2) (26). FANTOM5 was a source of CAGE-seq data for esophageal cancer cell line EC-GI-10 (27) while HACER: Human ACtive Enhancer was used to download global and precision nuclear run-on sequencing (GRO-seq and PRO-seq, respectively) data from HCT116 (28).

### 2.4 Quantitative Reverse Transcription PCR

Each cell line was collected for RNA extraction using an Illustra RNAspin kit. One hundred nanograms of total RNAs was reverse-transcribed by Quantabio cDNA synthesis reagents, and the prepared cDNAs were diluted and used for real-time PCR (RT-PCR) using customized or PrimePCR Array primers (Bio-Rad, CA). Each customized primer set (**Table S1**) was validated to generate a specific PCR product using melting curve analysis. To calculate normalized expression, the cycle threshold values of ACTB gene were used.

To measure the expression of ncRNAs, we performed digital droplet PCR (ddPCR, Bio-Rad) that has superior sensitivity to detect low gene expressions in samples with specifically designed primer sets (**Table S1**). To deplete any genomic DNA contamination, we extracted RNAs from cells with DNase digestion during RNA isolation (GE Healthcare) and confirmed that the quality of RNAs is satisfied by 260/280 ratio evaluation. Also, we treated DNase (EZ-DNase) again on the RNAs right before synthesizing cDNAs according to the manufacturer’s protocol (Thermo Fisher Scientific, MA).

### 2.5 Cell Viability Assay

Cells were seeded in 96-well plates at 5,000 cells per well and transfected with each antisense LNA-GapmeR (**Table S1**) for 48 h or treated with various concentration of T-5224 (5-[4-(cyclopentyloxy)-2-hydroxybenzoyl]-2-[(2,3-dihydro-3-oxo-1,2-benzisoxazol-6-yl)methoxy]-benzenepropanoic acid). Cell viability was evaluated using MTT solution [3-(4, 5-Dimethylthiazol-2-yl)-2,5-diphenyltetrazolium Bromide; Sigma-Aldrich] solution. For the assay, 20 µl of MTT solution (1 mg/ml) was added into each well containing 100 µl of media and cells, and the plate was placed in the incubator. After 3 h, all media, including MTT solution, were carefully removed, and 120 µl of DMSO (Macron Fine Chemicals, PA) was added to solubilize the purple precipitates. From each well, including the blanks, the absorbance at 570 nm was measured in a FlexStation 3 microtiter plate reader (Molecular Devices, CA). Relative cell number percentages were calculated by dividing the optical density of each group with the density of the control group, depending on experiments.

### 2.6 Western Blot

Whole cell proteins were extracted with RIPA lysis buffer (Thermo Fisher Scientific), and equal amounts of proteins were run in SDS-polyacrylamide gel, and the separated proteins were transferred to PVDF membranes. The blot was incubated with 5% nonfat dry milk blocking buffer (Bio-Rad) for 2 h and probed with specific antibodies for EGFR (Proteintech, IL) and α-Tubulin (Sigma-Aldrich) in 5% milk buffer overnight. After incubating the appropriate HRP-conjugated secondary antibodies (GE Healthcare, NJ), protein expressions were visualized using ECL detection methods (GE Healthcare) on ChemiDoc (Bio-Rad).

### 2.7 Scratch Wound Assay

Cells were seeded at a density of 2 × 10^5^/well in 12-well plates for 24 h, followed by being incubated in serum-free culture medium for another 12 h. A wound was then introduced by scraping a pipette tip across the monolayer and washed three times with PBS. Images of the scratch-treated cells were collected on microscope equipped with a 37°C and 5% cell incubator chamber.

### 2.8 Statistical Analysis

Results are presented as means ± SD unless otherwise indicated. Statistical analyses (*t*-test or one-way ANOVA followed by Tukey’s multiple comparison test) were performed using Prism 7 (GraphPad software, CA).

## 3 Results

### 3.1 ATAC-seq Revealed Distinct Chromatin Landscapes in Non-Cancerous Esophageal Epithelial Cells and ESCC Cells

To explore the characteristic changes in chromatin accessibility in ESCC, we performed ATAC-seq in two human ESCC cell lines, i.e., KYSE-30 and KYSE-150, and human non-tumorigenic esophageal epithelial cell line HET-1A. KYSE-150 is a poorly differentiated ESCC cell line with a fast proliferation rate, poor migration, invasion property, and amplified oncogene expression, such as *EGFR* (eightfold) and *CCND1* (fourfold). KYSE-30 cell line presents aggressive metastasis property with a 12-fold upregulated expression of EGFR (7, 29). HET-1A is widely used in esophageal cancer research as a reference non-tumorigenic cell line. It is derived from normal human esophageal cell with regular epithelial morphology and presents growth inhibition and differentiation under serum-supplemented media (30).

**Figure 1:**
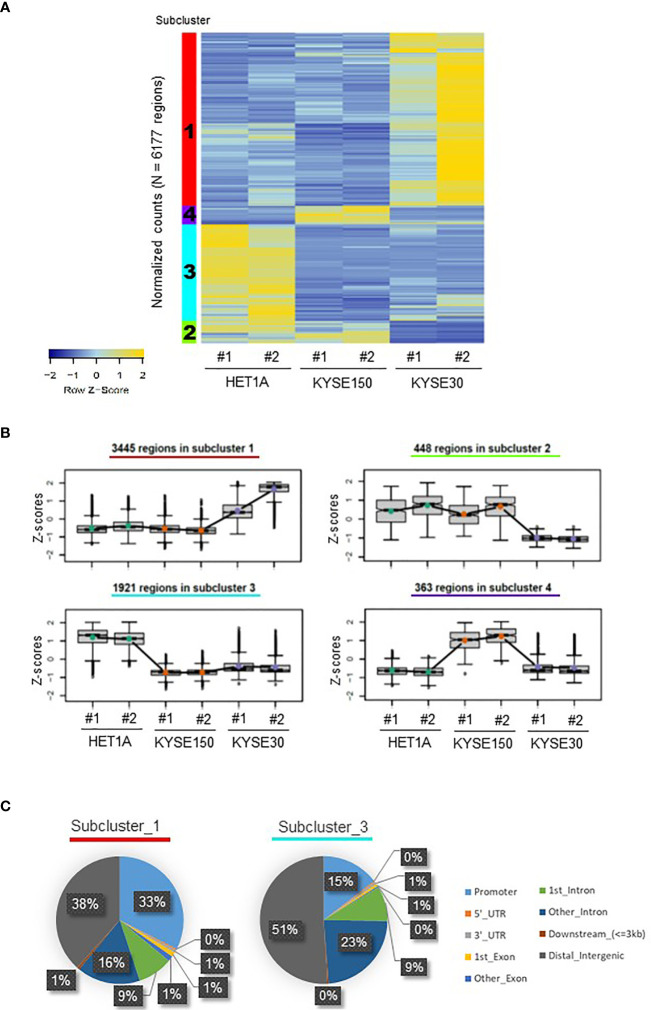
Distribution of ATAC-seq peaks in HET-1A, KYSE-150, and KYSE-30 cells. **(A)** A heatmap of differentially accessible chromatin regions in each sample, hierarchically clustered into four subclusters of hyper- or hypo-accessible regions among cell lines. The subcluster numbers are shown in the left color-coded columns. **(B)** Different trends of chromatin accessibility in total 6,177 regions among cell lines. The number of annotated regions in each subcluster is provided. **(C)** ATAC-seq peaks in subclusters 1 and 3 were differently distributed in the regulatory and non-regulatory genomic regions. Decimal numbers were omitted.

The qualities of ATAC-seq libraries were confirmed with Bioanalyzer analysis. Laddering pattern of DNA fragments ranging from small to large represents NFR and NCC nucleosomes (**Figures S1A, B**). As expected, ATAC-seq peaks from replicates were well correlated showing a high degree of concordance, and the open chromatin structure of HET-1A cells was disparate from that of the two ESCC cell lines (**Figure S1C**). For each sample, for further analysis, the DNA fragments were divided as NFR (sub-nucleosomal, <100 bp) and as NCC (>100 bp). The resultant NFR overlapping peaks consistent between replicates for the same cell line were further filtered for all peaks, which were consistently and significantly different (FDR < 0.05) between at least two cell lines. These were identified as differentially accessible DNA loci of ATAC-seq peaks in each cell line and hierarchically clustered resulting in their open or closed chromatin patterns (**Figures 1A, B**). Such a heatmap of cluster level aggregated accessibility patterns allowed for better representation of the chromatin changes in the samples. Each row in the heatmap corresponds to a differentially accessible location, and the colors represent the relative accessibility in the same. Subcluster 1 contains the most numbers of peaks (*n* = 3445 regions), and these are specifically accessible in KYSE-30 cells, suggesting that this aggressively tumorigenic cancer cell line presents more accessible chromatin structure than the other two cell lines. Subcluster 4 includes the differentially accessible regions (*n* = 363 regions) that are prominently found in KYSE-150 cells. On the other hand, subcluster 3 represents loci that are opened in the non-cancerous cell line and closed in both the cancer cell lines (*n* = 1,921 regions).

Next, we identified the locations of the differentially accessible peaks and the subclusters representing the distinct chromatin patterns (**Figures 1C** and **S1D**). Subclusters 1 and 4 containing groups of gene loci hyper-accessible in ESCC cells displayed locations in distal intergenic (38% and 34%, respectively) and promoter regions (33% for both). However, in subcluster 3, comprising a group of gene loci hyper-accessible only in HET-1A cells, the majority of accessible locations were found in istal intergenic (51%) and intronic regions (9% + 23% = 32%). Compared with subcluster 1, subcluster 3 particularly showed increased percentage of peaks in the second or more downstream intron regions and the decreased percentage of peaks in promoter. The different ATAC-seq peak distributions suggested that ESCC and non-cancer cells have very different chromatin structures. Both KYSE-30 and KYSE-150 cells have loose (open) chromatin in the promoter regions of various genes, which may support accessibility to transcription machinery and hence active transcription important for vigorous proliferation and metastasis, whereas non-cancer cells have a limited number of open promoter regions but relatively high portion of hyper-accessible introns and distal intergenic regions consistent with the need of tight regulation of gene expression.

### 3.2 Functional Enrichment Analysis of Hyper-Accessible Regions in KYSE-30 Cells or in HET-1A Cells

**Figure 2:**
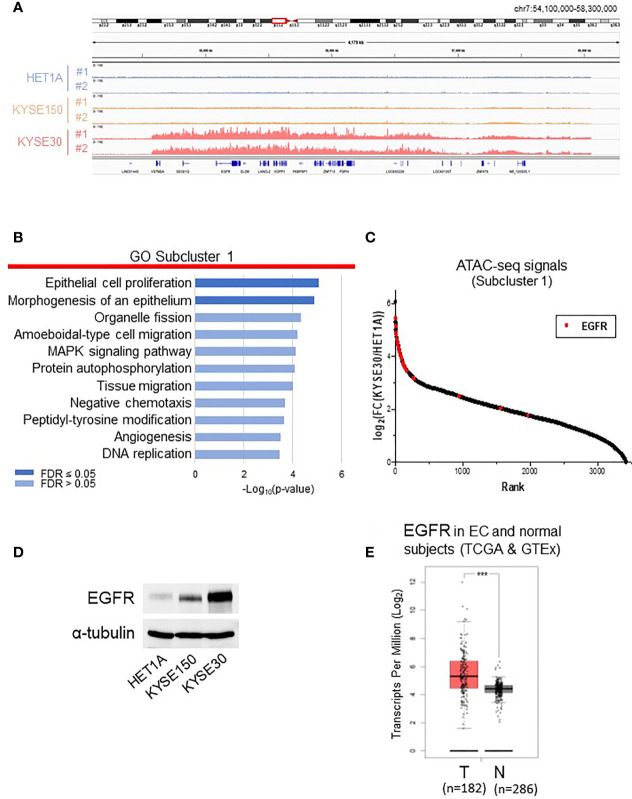
Analyses of subcluster 1 that includes highly accessible chromatin regions in KYSE-30 cells. **(A)** Exemplary ATAC-seq peaks on chromosome 7 showed that these regions are hyper-accessible in KYSE-30 cells. **(B)** Differentially accessible regions in subcluster 1 were associated to the nearest genes, and functional enrichment analysis of this gene set shows various cell signaling pathways associated with cancer. **(C)** In subcluster 1 ATAC-seq peak set (*n* = 3445 peaks), the fold changes of peak frequencies in KYSE-30 versus HET-1A cells were calculated and ranked. The highest number of differential ATAC-seq peaks were found near the EGFR gene, and most of these peaks (28/31) were ranked within the top 10%. Red marks indicate the fold change of each EGFR-associated ATAC-seq peak (*n* = 31). **(D)** Representative Western blot showed that EGFR expression was upregulated in KYSE-150 and KYSE-30 cells. **(E)** EGFR gene had higher expression in tumors (T) than in neighboring tissues of esophageal cancer patients (TCGA data) or normal esophagus (GTEx data) (N). T = 182, N = 286; Log2FC >0.5; ***p-value < 0.001.

To compare the chromatin structures of non-tumorigenic HET-1A cells and cancer cells with aggressively tumorigenic and metastatic nature, we focused on analyzing subclusters 1 and 3, which contain the majority of differentially accessible ATAC-seq peaks (**Figures 2** and **S2**). KYSE-30 cells presented much more ATAC-seq peaks, for instance, on chromosome 7, indicating that these regions are hyper-accessible in this metastatic cell line (**Figure 2A**). We annotated the peaks in the subclusters to the nearest genes and performed gene enrichment analysis using the WebGestalt tool (18) with enrichment categories from the databases GO (Biological Process) (19, 20), KEGG (21), Reactome (22), Wikipathways (cancer) (23), and Disgenet (24) (**Figures 2B**, **S2B** and **Table S4**). In subcluster 1, the two most significantly enriched GO terms were “epithelial cell proliferation” and “morphogenesis of an epithelium”, which are consistent with KYSE-30 cells presenting fast proliferation and squamous epithelial morphogenesis.

Then, in subcluster 1, the fold change of each peak frequency in KYSE-30 versus HET-1A cells was calculated and ranked (**Figure 2C** and **Table S2**). Interestingly, we found that EGFR gene locus has the greatest number of differential ATAC-seq peaks and that majority of EGFR-associated peaks show high abundance in KYSE-30 cells compared to the HET-1A cells. This chromatin landscape in EGFR locus is consistent with massive expression of EGFR protein in KYSE-30 cells compared to HET-1A and KYSE-150 cells (**Figure 2D**). RNA-seq data from TCGA database confirmed that EGFR expression is indeed significantly higher in tumors than that in adjacent non-tumor tissues in esophageal cancer patients (**Figure 2E**). As such, we postulated that overexpression of EGFR observed in human esophageal cancer cells or tumor tissues is due to hyper-accessibility of chromatin structure near EGFR gene locus in cancer cells.

### 3.3 ATAC-seq Peaks Revealed Non-Coding RNAs Near EGFR Gene Locus

**Figure 3:**
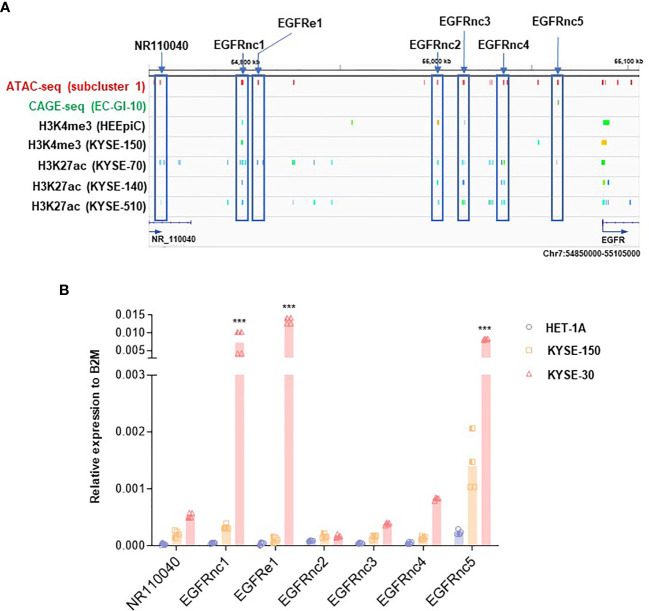
Identification of non-coding RNAs (ncRNAs) near EGFR upregulated in KYSE-30 cells. **(A)** Multi-omics analysis approach revealed non-coding RNAs upstream of EGFR that are overlapped with both hyper-accessible regions in KYSE-30 cells (ATAC-seq peaks) and potential enhancer regions in other ESCC cells (CAGE-seq or H3K27ac peaks). Various colors in each ChIP-seq row indicate different peaks that are generated from one sequencing result. Blue boxes highlight the non-coding RNAs tested in this study. **(B)** Expressions of ncRNAs in HET-1A, KYSE-150, and KYSE-30 cells were measured by digital droplet PCR, and their copy numbers relative to the copy numbers of B2M, a commonly used housekeeping gene, are shown. Two technical replicates from two biological replicates are displayed. ***p-value < 0.001 compared to each gene in HET-1A cells.

We then examined ATAC-seq peaks in subcluster 1 located in the upstream of EGFR locus in KYSE-30 cells with higher resolution (**Figure 3A**). Compared with datasets from CAGE and ChIP-seq for H3K27ac in ESCC cells (26, 27), we found that the hyper-accessible regions in KYSE-30 cells found in our ATAC-seq data overlap well with regions identified by these datasets, suggesting the possible existence of active enhancers in those regions in ESCC. In addition, we investigated transcriptionally active ncRNAs by referring to the ChIP-seq of RNAP II, GRO-seq, and PRO-seq near EGFR locus (26, 28) and found that these estimated-RNAP II-driven ncRNAs in the EGFR upstream region are well-overlapped with our ATAC-seq data (**Figure S3A**). Since RNAP II-binding regions in intergenic areas may represent the transcription of ncRNAs (31), we named these estimated ncRNAs as EGFRnc1-5 and EGFRe1. The EGFRnc1 and EGFRe1 are located at 27.0 kb and 34.5 kb downstream from NR_110040, respectively. Interestingly, both regions were detected as ATAC-seq peaks in tumor tissues removed from esophageal cancer patients (**Figure S3B**; ATAC-seq dataset of TCGA esophageal cancer patient samples was obtained from https://atacseq.xenahubs.net) (32).

To evaluate if these EGFR-upstream regions indeed generate ncRNAs, we synthesized cDNAs from RNAs with double-DNase treatments from each cell line and performed ddPCR known to be capable of detecting very low expressed genes (33). EGFRnc1-5 and EGFRe1 expression levels from all three cell lines were higher than the level of the validated transcript ”NR110040” of HET-1A cells, which indicates that EGFRnc1-5 and EGFRe1 are indeed transcribed into ncRNAs (**Figure 3B**). Interestingly, all determined ncRNAs showed trends of increase in ESCC cells compared with those in HET-1A cells, which is in accordance with the higher expression of EGFR in ESCC cells. Among these ncRNAs, EGFRnc2 expression was the lowest and EGFRe1 and EGFRnc5 were the two most abundant in KYSE-30 cells compared to those in HET-1A cells, with over 300- and 30-fold increase, respectively (**Figure S3B**).

### 3.4 EGFRe1, a Putative Enhancer RNA for EGFR Gene, Regulated Cell Proliferation and Migration in ESCC Cells

**Figure 4:**
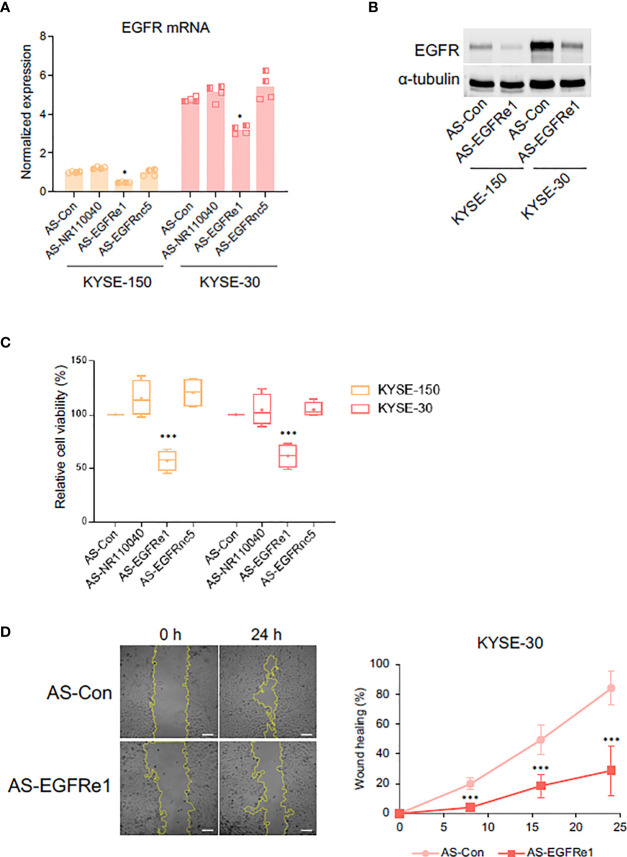
Impact of EGFRe1, a putative enhancer RNA for EGFR, on cell proliferation and migration of KYSE-30 cells. **(A)** EGFR mRNA expression was examined in cells transfected with each LNA-AS targeting control or ncRNAs. Normalized expression was obtained from comparing relative expression (EGFR/Actb) in each group to that in the KYSE-150 control group. Two technical replicates from two biological replicates were displayed. *p-value < 0.05 compared to group of control LNA-AS transfection (AS-Con). **(B)** Representative Western blot showed that suppressing EGFRe1 reduced EGFR expression in both KYSE-150 and KYSE-30 cells. **(C)** Cell viability rate in each LNA-AS-transfected group was compared to the AS-Con group in each cell line. The AS-Con group was used as 100%. Box-and-whisker plots were drawn with four biological replicates, and a plus sign in each box represents the mean value. ***p-value < 0.001 compared to AS-Con in each cell line. **(D)** Wound healing rates in AS-Con and AS-EGFRe1 transfected KYSE-30 cells were plotted in the indicated post-wound-time points (right). Representative phase contrast images at 0 h and 24 h are displayed (left). ***p-value < 0.001 compared to AS-Con in each time point.

Enhancer-derived ncRNA (eRNA) is known both to positively correlate with mRNA expression of nearby genes and to contribute to the mRNA transcription (34). To determine whether EGFRe1 and EGFRnc5 are eRNAs regulating EGFR gene expression, we knocked down their gene expression using specifically designed locked nucleic acid antisense oligonucleotides (LNA-ASs) and examined EGFR mRNA expression (**Figures 4A** and **S4**). The reduced level of NR110040 and EGFRnc5 transcripts did not affect EGFR mRNA expression in both KYSE-150 and -30 cells. However, EGFRe1 knockdown in both cancer cell lines significantly downregulated EGFR mRNA expression, compared to control LNA-AS transfected cells. Corresponding to its mRNA expression, EGFR protein expression was also decreased by EGFRe1 knockdown in both ESCC cell lines (**Figure 4B**).

Next, we tested whether EGFRe1 knockdown has any impact on ESCC cell proliferation and migration by alleviating EGFR expression. As shown in **Figure 4C**, EGFRe1 knockdown reduced cell proliferation rates in KYSE-150 and KYSE-30 cells by 45% and 40%, respectively, while NR110040 or EGFRnc5 knockdowns had no impact on cell proliferation. Cell migration rates of KYSE-30 cells were measured by wound healing assay. EGFRe1 knockdown decreased cell migration evidenced by significant reduction of wound healing from 8 h post-wounding (**Figure 4D**). All these data suggest that EGFRe1 is a putative EGFR eRNA and contributes to EGFR overexpression and subsequently cell proliferation and migration in ESCC cells.

### 3.5 Transcription Factor Binding Motif Analysis Identified AP-1 Family at Open Chromatin Regions in ESCC Cells

**Figure 5:**
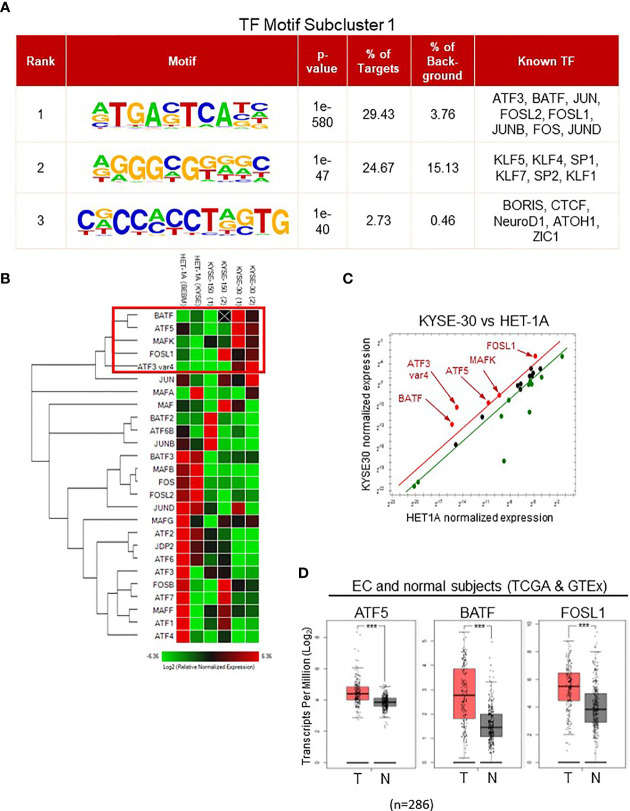
Identification of AP-1 family in subcluster 1 by transcription factor motif analysis. **(A)** All ATAC-seq peaks in subcluster 1 were analyzed, and the potential transcription factors bound to the DNA and their sequence motifs were displayed in rank. **(B)** PCR array with probes targeting all AP-1 family member was performed. Heatmap displayed the RNA expression-based clustering of AP-1 family genes in HET1A, KYSE150, and KYSE30 cells. Genes in a red rectangle represented the upregulated genes in KYSE30 cells. HET-1A cells were incubated in the BEBM base medium [HET-1A (BEBM)] or identical medium for KYSE cells [HET-1A (KYSE)] overnight before RNA collection while KYSE-150 and -30 cells were cultured in identical conditions. **(C)** Significantly upregulated genes in KYSE-30 (red) versus HET-1A cells are shown in red. Green dots represent the downregulated genes. **(D)** ATF5, BATF, and FOSL1 gene expressions in tumors (T) were higher than that in neighboring tissues of EC patients or normal esophagus (N). T = 182, N = 286; Log2FC >0.5; ***p-value < 0.001.

Given that open chromatin regions contain the cis-regulatory modules with specific sequences for the binding of particular transcription factors (6), examining recurrent motifs in hyper-accessible regions allowed us to estimate the transcription factors involved in each subcluster (**Figures 5A** and **S5**). In subcluster 1, the most frequently found motifs contained the core sequence TGA(C/G)TCA. This sequence is the specific recognition site for AP-1 transcription factor family including ATF3, BATF, JUN, and FOSL2. Data implied that AP-1 members are potential regulatory factors involved in regulating hyper-accessible chromatin structure in KYSE-30 cells.

To examine which AP-1 members were involved in regulating chromatin structure in ESCC cells, we determined the gene expressions of various AP-1 transcription factors in HET-1A and ESCC cells (**Figures 5B, C**). Among 25 AP-1 members and a gene variant, only BATF, ATF5, MAFK, FOSL1, and ATF3 variant 4 were upregulated in KYSE-30 cells compared to HET-1A cells. Interestingly, majority of ATF3 transcripts were decreased in KYSE-30 cells whereas ATF3 variant 4, a variant without DNA binding motif, was increased by more than 20 times (**Figure S6**). The TCGA RNA-seq database for esophageal cancer patients showed higher expression of ATF5, BATF, and FOSL1 in cancer tissues, which is consistent with the finding of greater expression of these genes in KYSE-30 cells (**Figure 5D**).

### 3.6 A Selective AP-1 Inhibitor Alleviated Cell Proliferation and Migration in KYSE-30 Cells

**Figure 6:**
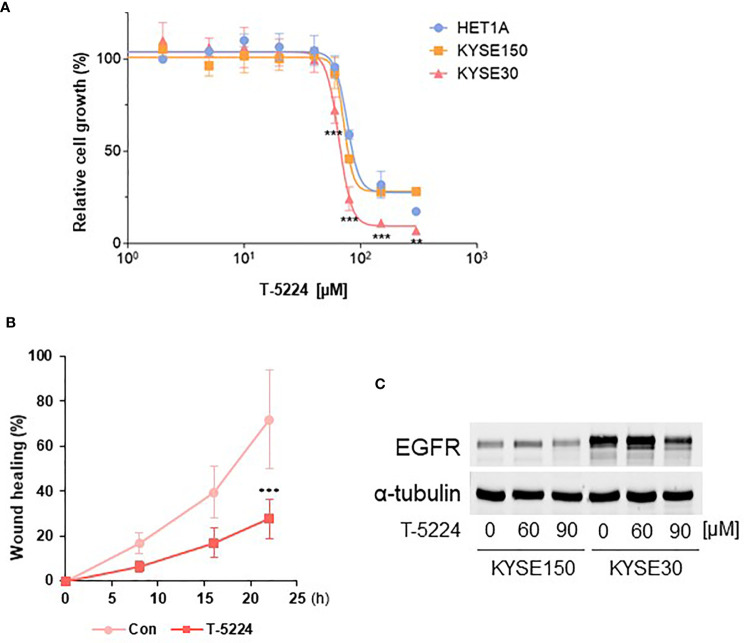
Inhibition of proliferation and migration of KYSE-30 cells by AP-1 inhibitor T-5224. **(A)** Cell viability rates upon different doses of T-5224 for 24 h were measured with MTT assay. Data are shown as nonlinear fit of log-dose *vs.* response curves. Error bars represents mean ± SD from three independent experiments. ***p-value < 0.001, **p-value < 0.01 compared to HET-1A at each time point. **(B)** Wound healing rate of KYSE-30 cells upon 60 μM T-5224 treatment was plotted in the indicated post-wound time points. ***p-value < 0.001 compared to control (DMSO-treated) in each time point. **(C)** Representative Western blot shows that EGFR expression decreased by T-5224 in KYSE-30 cells. Indicated concentrations of T-5224 were treated for 24 h in both cell lines.

Based on the finding that AP-1 binding sites are broadly opened in KYSE-30 cells, we postulated that inhibiting AP-1-DNA binding may reduce the cancer cell characteristics, such as proliferation and migration, of KYSE-30 cells. Using cell viability assay, we found that the KYSE-30 cell line is significantly more sensitive to T-5224, an AP-1 inhibitor. The IC50 of T-5224 was significantly lower in KYSE-30 cells (65.2 µM) than that in KYSE-150 (72.1 µM) and HET-1A (76.7 µM) cells (**Figure 6A**). The inhibitor also reduced cell migration of KYSE-30 cells (**Figure 6B**).

Since the upstream of EGFR gene contains certain AP-1 factor binding motifs, we sought the effect of an AP-1 inhibitor on EGFR expression in ESCC cells. Treatment of 90 µM T-5224 induced more than 50% reduction of EGFR expression in KYSE-30 cells (**Figure 6C**), consistent with significantly reduced cell viability (**Figure 6A**). Corresponding to this, ChIP-seq datasets of BATF, MAFK, and FOSL1 showed that these AP-1 members may bind to EGFR upstream regions, which are overlapped with our ATAC-seq peaks (**Figure S7**). Taken together, these data suggest that AP-1 transcription factors could regulate chromatin EGFR locus and thus control cell proliferation and migration in ESCC cells.

## 4 Discussion

During carcinogenesis and tumor development, cells undergo drastic genetic and epigenetic alterations that are reflected by distinct chromatin landscapes in cells at each stage. In this study, we performed ATAC-seq and compared the regulatory chromatin landscapes among KYSE-150 and KYSE-30, two human ESCC cell lines and HET-1A, a non-tumorigenic esophageal epithelial cell line. The data revealed distinct hyper-accessible regions in each cell line. In one of the hyper-accessible gene loci in KYSE-30 cells, we found a ncRNA in the upstream of EGFR locus as a putative eRNA. Further motif analysis, gene knockdown and pharmacological data support that AP-1 family transcription factors can bind the hyper-accessible regions and affect their proliferation and migration. Altogether, this study explored the chromatin accessibility landscapes based on ATAC-seq assay and discovered a new eRNA for EGFR gene in ESCC and the reliance of cancer cells on the AP-1 transcription factor.

Three cell lines were selected for ATAC-seq analysis, i.e., HET-1A, KYSE-150, and KYSE-30, respectively representing non-cancerous cells, cancer cells with rapid proliferation but relatively lower migration/invasion property, and cancer cells with aggressive metastatic characteristics (7, 11, 30, 35, 36). As expected, HET-1A cells contained different chromatin structures from KYSE-150 or KYSE-30 cells. In subcluster 3, HET-1A cells presented more hyper-accessible regions (**Figures 1B**, **S2**, and **Table S4**). Functional enrichment analysis showed that this subcluster contained many known genes related to extracellular structure organization, cell-substrate adhesion, and junction organization. Interestingly, many other genes in this subcluster have not been associated with maintaining esophageal epithelia or cancer prevention function. For example, genomic regions near ZNF536 were hyper-accessible in HET-1A cells but less-accessible in both ESCC cell lines, which aligns with observation of significantly lower ZNF536 expression in human esophageal tumor tissues (**Figure S2**). While further study is required to examine the exact function of this gene in ESCC, nevertheless, chromatin accessibility obtained by ATAC-seq analysis can provide rich data to reveal novel loci or genes involved in carcinogenesis and tumor development.

KYSE-30 and KYSE-150 cells appeared to have hyper-accessible chromatin regions in subclusters 1 and 4, respectively (**Figure 1**). Again, the functional enrichment analysis provided guidance to identify new loci or genes involved in ESCC. For example, the promoter regions of genes involved in histone gene cluster 1 are more accessible in KYSE-150 cells, suggesting that these cell cycle-dependent genes are highly activated to support cancer cells’ rapid proliferation (data not shown). In subcluster 2, KYSE-30 contained hypo-accessible chromatin regions (**Figure 1B**). The different hyper- or hypo-accessible chromatin regions between KYSE-150 and -30 cells suggest heterogeneities and divergences in oncogene activations, carcinogenesis, and cell behaviors in different ESCC cells. In the present study, we focused on subcluster 1, which contains the most number of ATAC-seq peaks and represents hyper-accessible genomic regions in KYSE-30 cells. Although it was originally isolated from a well-differentiated tumor tissue and known to induce medium-differentiated tumor in nude mice, the KYSE-30 cell line is known to aggressively migrate and metastasize as its primary tumor was found to invade contiguous structures (29). Given that migrating cancer cells are more resistant to chemo- and radiotherapy than non- or restricted-migrating cells (37), ATAC-seq peaks in subcluster 1 may contain many characteristic open chromatin regions associated with these resistances. Further investigation of these chromatin regions is warranted.

Using multi-omics approach, we identified six ncRNAs upstream to EGFR gene locus (from −186,000 bp to −23,000 bp from EGFR transcription start site). Their existence was estimated from various genomic sequencing data that we employed, including accessibility of chromatin (ATAC-seq), enrichments of specific histone modifications and RNAP II binding (H3K27ac and RNAP II ChIP-seqs), and locations of actively transcribing RNA polymerases (GRO and PRO-seqs) and/or transcription start site (CAGE). Given that some of these datasets have often been used to estimate active enhancers on intergenic regions and that the closest downstream gene locus from these ncRNAs is EGFR, they were expected to be eRNAs positively regulating EGFR expression. These open chromatin regions were also found in tumor samples extracted from esophageal cancer patients (**Figure S3**). EGFRnc2 was not correlated with EGFR expression in ESCC cells, and EGFRnc5 was not responsible for EGFR mRNA expression according to our gene silencing experiment. These findings suggest that genomic data-estimated eRNA should be functionally validated by genetic perturbation experiments on the candidate RNA, in order to prove that it indeed is an eRNA. EGFRnc1 was not functionally tested here, but its role as an eRNA, to regulate either the expression of EGFR alone or multiple genes is worth to be examined in the future due to its high expression in ESCC cells and high frequency of overlap in the above genome data sets. In addition, two important features of eRNAs, i.e., the length and the direction of the transcripts, require further investigation using appropriate techniques, such as RNA-Seq/PRO-Seq/5’GRO-Seq. To our knowledge, this is the first report suggesting EGFRe1 as an eRNA for EGFR, which can regulate EGFR overexpression in ESCC cells. Previously, using ChIP-seq analyses of H3K4me3 and H3K27AC, a “super enhancer” of EGFR was identified in cervical cancer cells (38). The super enhancer produced the RNA transcript and positively associated with EGFR mRNA expression. Although it was not described in detail, according to the fact that it is localized within the first intron and detected by specific PCR primers, the super enhancer may span EGFRint1, 2, 3, and 4 in our data. Since all these intronic RNAs are upregulated in both ESCC cell lines, their possible roles in EGFR gene in ESCC, as in cervical cancer, need to be further investigated.

Suppressing EGFRe1 not only decreased EGFR expression, but also inhibited both proliferation and migration in ESCC cells. Current EGFR targeting therapy using either TKIs or antibodies did not show expected therapeutic benefits for esophageal patients, and EGFRe1-dependent inhibitory function may have potential values. In particular, while gefitinib, a first-generation TKI, was effective only on esophageal cancer patients with *EGFR* copy number gain (39), EGFRe1 silencing is effective in both KYSE-30 and -150 cells, which suggests that inhibiting EGFRe1 can be considered to treat a wider spectrum of ESCC types with high EGFR levels obtained not only by gene copy number gain but also by protein overexpression. Pre-clinical models with gene suppression techniques such as antisense oligonucleotides, siRNA, and CRISPR interference, will provide further evaluate EGFRe1 as a new therapeutic measure for ESCC.

The critical roles of AP-1 in esophageal cancer have been previously suggested (40, 41). Using DNA binding motif analysis, we identified AP-1 as a critical transcription factor regulating chromatin dynamics in ESCC. In addition to its conventional roles as downstream or upstream players of MAPK and other inflammatory signaling, recent genome-wide studies have revealed new roles of the AP-1 family in chromatin accessibility functioning in both proximity and remoteness on their target promoters and distal enhancers, respectively (42, 43). For instance, AP-1 was identified as a regulator of genome-wide open chromatin structure and gene transcription profiles in EAC (40). Our ATAC-seq data showed an open chromatin region about −500 bp upstream of EGFR TSS (**Figure S7**, far right red box), which is anticipated to bind FOSL1, an AP-1 family member, according to publicly available ChIP-seq data. In addition to this reported AP-1 binding site near the promoter region, we also found more hyper-accessible regions at remote regions upstream of EGFR TSS, which can largely overlap with ChIP-seq data of FOSL1, BATF and MAFK (3 AP-1 family members) (**Figure S7**). The data suggest that these regions could contain putative AP-1 binding sites. Therefore, ATAC-seq appears to be a good tool to study a transcription regulatory protein’s multiple functions. It may reveal not only local transcriptional switch functioning in the proximity of TSS, but also remote controller though binding to distal enhancers, placing chromatin architecture dynamics to regulate the overall transcriptional actions. These information may assist the understanding of novel mechanisms underlying fine-tuned gene regulations in the context of chromatin 3D organization and architecture. Since transcription factor-mediated gene transcription depends on both its accessibility to cognate binding sites and its abundance, the authors determined gene expression of JUN and FOS family members and found overexpression of FOS, FOSB, and FOSL1 in the cancer. In ESCC cell lines, we examined all AP-1 transcription factors including JUN, FOS, MAF, and ATF family members and found that FOSL1, MAFK, BATF, and ATF5 are upregulated compared to non-cancer cells. These data suggest that, although AP-1 binding sites are hyper-accessible in both EAC and ESCC, specific AP-1 members bound to the sites forming hetero- or homo-dimer would vary. Interestingly, FOSL1 is a common AP-1 member highly expressed in both EAC and ESCC (6). Its overexpression and critical roles in cell proliferation and migration were also found in other squamous cell carcinoma, such as skin, head, and neck cancers (44). The exact mechanism underlying the modulatory function of FOSL1 in cancer-related cell signaling, oncogene transcription, and cancer-specific chromatin structure forming DNA-loop requires further extensive investigation.

## 5 Conclusions

Using the ATAC-seq approach, this study identified a putative eRNA for EGFR in hyper-accessible regions by analysis of chromatin accessibility landscapes and discovered the reliance on AP-1 transcription factor in ESCC cells.

## Data Availability Statement

The datasets presented in this study can be found in online repositories. The names of the repository/repositories and accession number(s) can be found in the article/**Supplementary Material**.

## Author Contributions

Conceptualization, ZP and SC. Methodology, ZP and SC. Formal analysis, AS, EM, CX, and SC. Investigation, SC and AS. Writing, CS, SC, ZP, AS, and EM. Visualization, SC and AS. Supervision, ZP and CX. Project administration, ZP. Funding acquisition, ZP. All authors contributed to the article and approved the submitted version.

## Funding

This work was supported by U.S. National Institutes of Health (NIH) Grant R01 CA185055 and S10 OD025230 (to ZP).

## Conflict of Interest

The authors declare that the research was conducted in the absence of any commercial or financial relationships that could be construed as a potential conflict of interest.

## Publisher’s Note

All claims expressed in this article are solely those of the authors and do not necessarily represent those of their affiliated organizations, or those of the publisher, the editors and the reviewers. Any product that may be evaluated in this article, or claim that may be made by its manufacturer, is not guaranteed or endorsed by the publisher.

## Supplementary Material

Supplementary Table S1 Sequences of customized primer and antisense LNA-GapmeR sets.

Supplementary Table S2 Gene ranking in each subcluster in ATAC-seq data.

Supplementary Table S3 Differential peaks in each subcluster.

Supplementary Table S4 Gene enrichment analysis for each subcluster.

Supplementary Figure S1 Evaluation of quality of ATAC-seq libraries. **(A)**. Bioanalyzer gel images of DNA libraries from all cell replicates show the unique laddering patterns of ATAC-seq libraries. **(B)**. A representative Bioanalyzer image displays that Tn5 transposase reaction occurred according to chromatin sizes. **(C)**. Correlation plot shows the differences among cells and correlation between replicates. **(D)**. ATAC-seq peaks in subcluster 4 are distributed on the genome similar to subcluster 1 while subcluster 2’s are unique compared to others. Decimal numbers have been omitted.

Supplementary Figure S2 Analyses of subcluster 3 with highly accessible chromatin regions in HET-1A cells. **(A)**. Exemplary ATAC-seq peaks on chromosome 19 show hyper-accessible regions in HET-1A cells but not ESCC cells. **(B)**. Gene ontology (GO) analysis of the differentially accessible gene set shows various cell signaling pathways associated with histone and chromatin structures. **(C)**. In subcluster 3 ATAC-seq peak set (n = 1923 peaks), the fold changes of peak abundances in HET-1A versus KYSE-30 cells were calculated and ranked. Most of peaks were highly ranked, representing significantly different ATAC-seq signals between HET-1A and KYSE-30 cells. Cyan marks indicate the each ZNF-536-associated ATAC-seq peak (n = 62 peaks). **(D)**. ZNF536 gene expression is lower in tumors (T) than that in neighboring tissues of esophageal cancer patients or normal esophagus (N). T=182, N=286; Log2FC >0.5; *** p-value < 0.001.

Supplementary Figure S3 Identification of ncRNAs near or within EGFR locus that were up-regulated in KYSE30 cells. **(A)**. ATAC-seq subcluster 1 peaks were compared to CAGE of normal esophageal epithelial tissues, GRO/Pro-seq/CAGE of HCT116, RNAP II ChIP-seq of digestive tract cells, and histone ChIP-seq of esophageal cell lines. Overlapped peaks are highlighted in blue (NR110040 intronic ncRNA and 34 other intergenic ncRNAs) and green boxes (EGFR intronic ncRNAs and EDLR ncRNA). The EGFRnc1 and EGFRe1 are located 27.0kb and 34.5 kb downstream from NR_110040, respectively. **(B)**. Tumor tissues removed from esophageal cancer patients revealed similar ATAC-seq peaks in the NR110040 and EGFR regions. The ATAC-seq dataset of TCGA esophageal cancer patients samples was obtained from https://atacseq.xenahubs.net. **(C)**. Normalized expressions of EGFRe1 and EGFRnc5, each gene’s expression in HET-1A being regarded as 1, in HET-1A, KYSE-150 and KYSE-30 cells were calculated from ddPCR data. Two biological replicates with two technical replicates were used to draw bar graphs. *** p-value < 0.001 compared to each gene in HET-1A cells. **(D)**. Expressions of ncRNAs in green boxes in HET-1A, KYSE-150, and KYSE-30 cells were measured by digital droplet PCR, and their copy numbers relative to B2M gene numbers are shown. Two biological replicates with two technical replicates were used to draw bar graphs. *** p-value < 0.001 compared to each gene in HET-1A cells; ^ǂǂ^ p-value <0.01 comparison of two different genes in KYSE-30 cells.

Supplementary Figure S4 Reduced expression of ncRNAs by specifically designed LNA-ASs. KYSE-150 and KYSE-30 cells were transfected with LNA-ASs for NR110040, EGFRe1, EGFRnc5 genes, or non-target sequence, and the respective ncRNA expression was measured by qRT-PCR. Normalized values with B2M control gene expression are shown. Two biological replicates with two technical replicates were used to draw bar graphs. ** p-value < 0.01 compared to each gene in HET-1A cells.

Supplementary Figure S5 Transcription factor motif analyses in each subcluster. All ATAC-seq peaks in each subcluster were examined for DNA motifs using HOMER [41], and the potential transcription factors bound to the DNA and their sequence motifs are displayed in rank.

Supplementary Figure S6 ATF3 expression in ESCC cell lines and esophageal cancer tissue. **(A)**. Primers were esigned for detecting all isoforms of ATF3 gene (ATF3) or specifically variant 4 isoform (ATF3 var4) and utilized to conduct qRT-PCR with indicated cell lines. While ATF3 expression is not different among cell lines, ATF3 variant 4 expression is significantly higher in KYSE-30 cells. Two biological replicates with two technical replicates were used to draw bar graphs. *** p-value < 0.001 compared to expression in HET-1A cells. **(B)**. ATF3 gene expression in tumors (T) is higher than in neighboring tissues of EC patients 35 and normal esophagus (http://gepia2.cancer-pku.cn). ATF3 gene isoform information in the RNA-seq data was not available. (N). T=182, N=286; Log2FC >0.5; *** p-value < 0.001.

Supplementary Figure S7 Comparison of ATAC-seq subcluster 1 peaks with AP-1 family member binding sites. Red boxes highlight the over-lapped regions between ATAC-seq subcluster 1 peaks and ChIP-seq datasets of BATF, MAFK, and FOSL1 in digestive tract cells, suggesting that, in KYSE-30 cells, these AP-1 family members possibly bind to these regions.
