# Supplementary material for: Identification of a Putative Enhancer RNA for EGFR in Hyper-Accessible Regions in Esophageal Squamous Cell Carcinoma Cells by Analysis of Chromatin Accessibility Landscapes
Source: Front Oncol. 2021 Oct 15;11:724687. doi: 10.3389/fonc.2021.724687 (PMC8554337; doi:10.3389/fonc.2021.724687)
Supplement: Supplementary Figure S1 — Evaluation of quality of ATAC-seq libraries. (A). Bioanalyzer gel images of DNA libraries from all cell replicates show the unique laddering patterns of ATAC-seq libraries. (B). A representative Bioanalyzer image displays that Tn5 transposase reaction occurred according to chromatin sizes. (C). Correlation plot shows the differences among cells and correlation between replicates. (D). ATAC-seq peaks in subcluster 4 are distributed on the genome similar to subcluster 1 while subcluster 2’s are unique compared to others. Decimal numbers have been omitted. [file DataSheet_1.pdf]

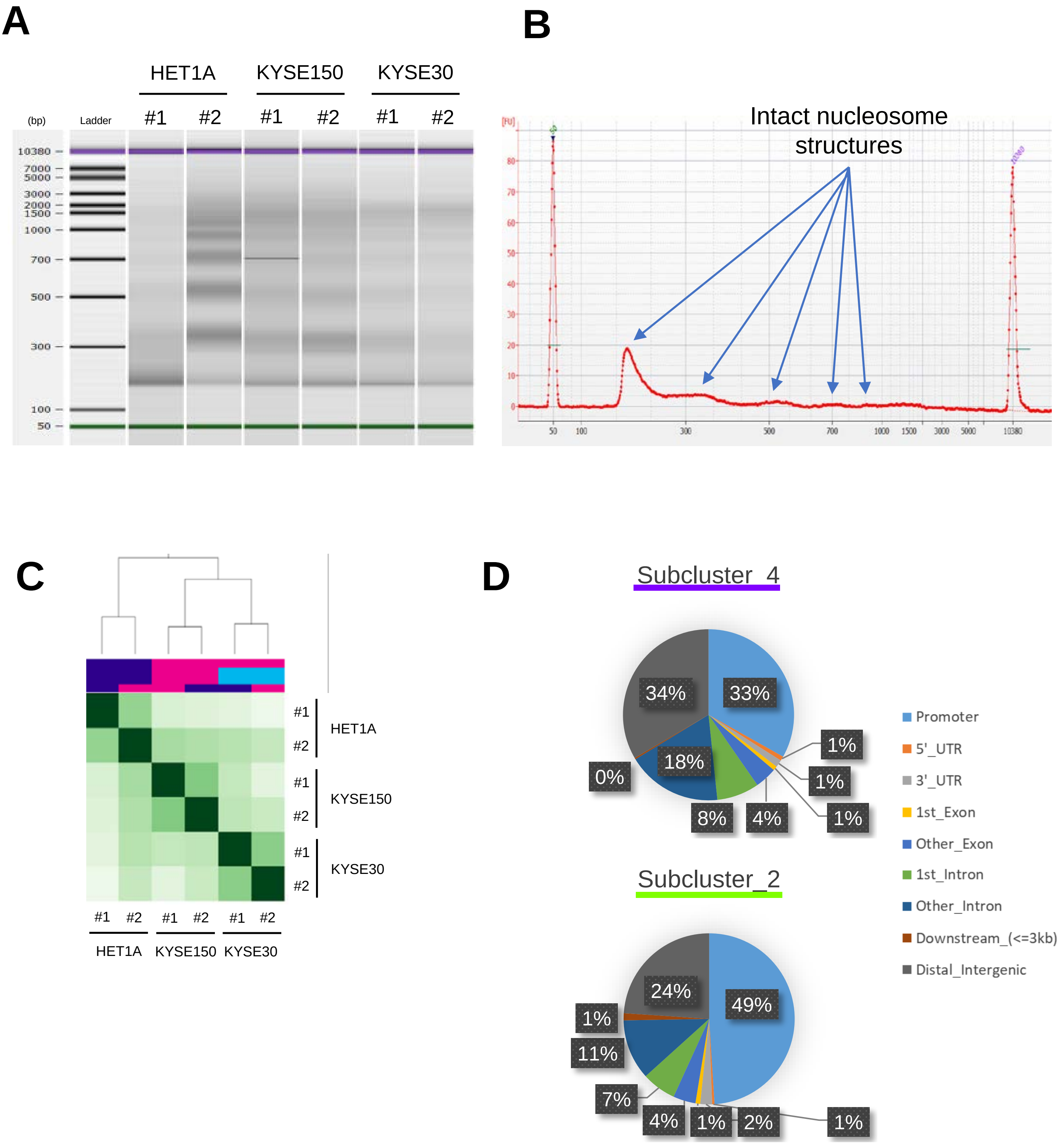

Choi, et al. Figure S1

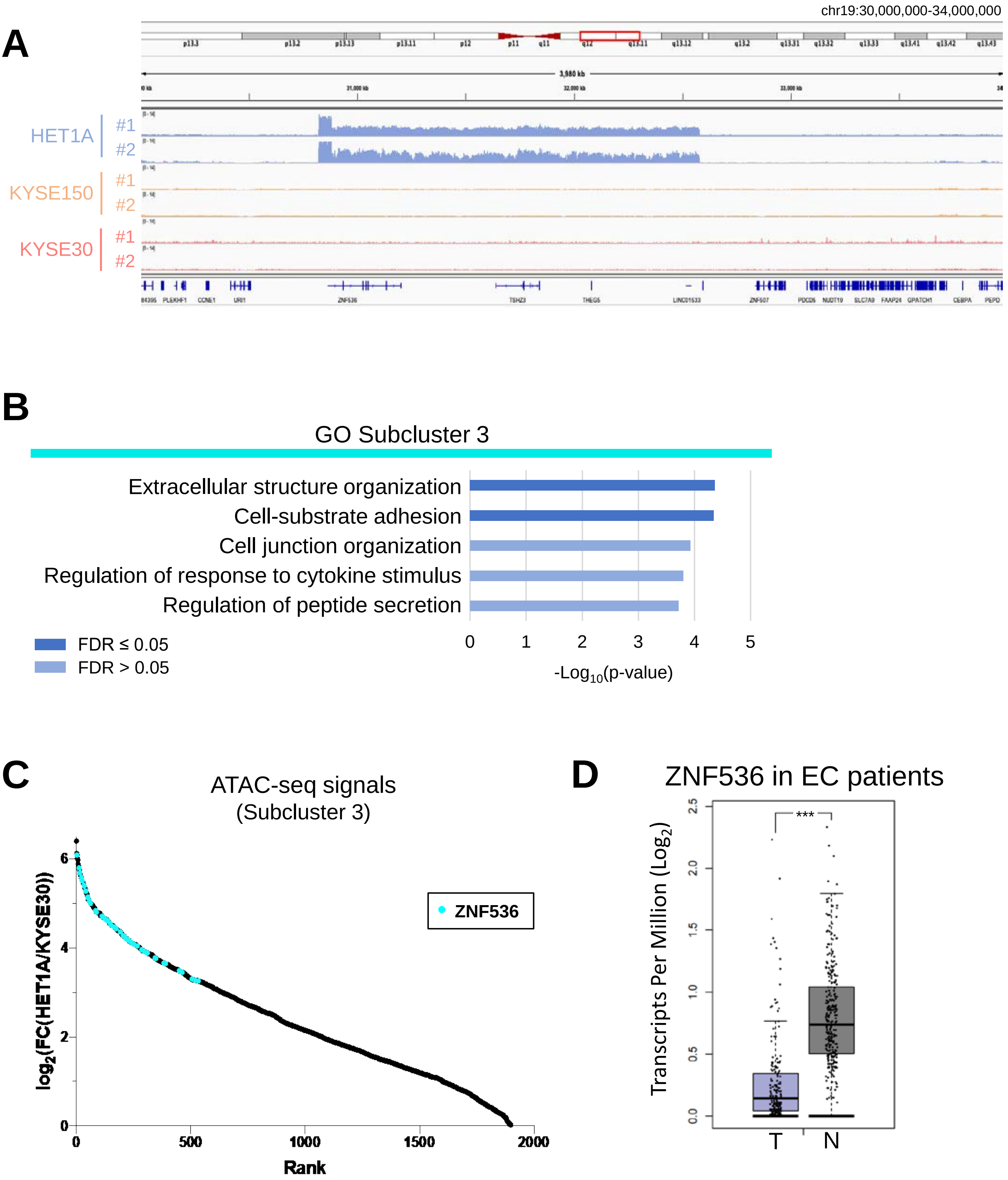

Choi, et al. Figure S2

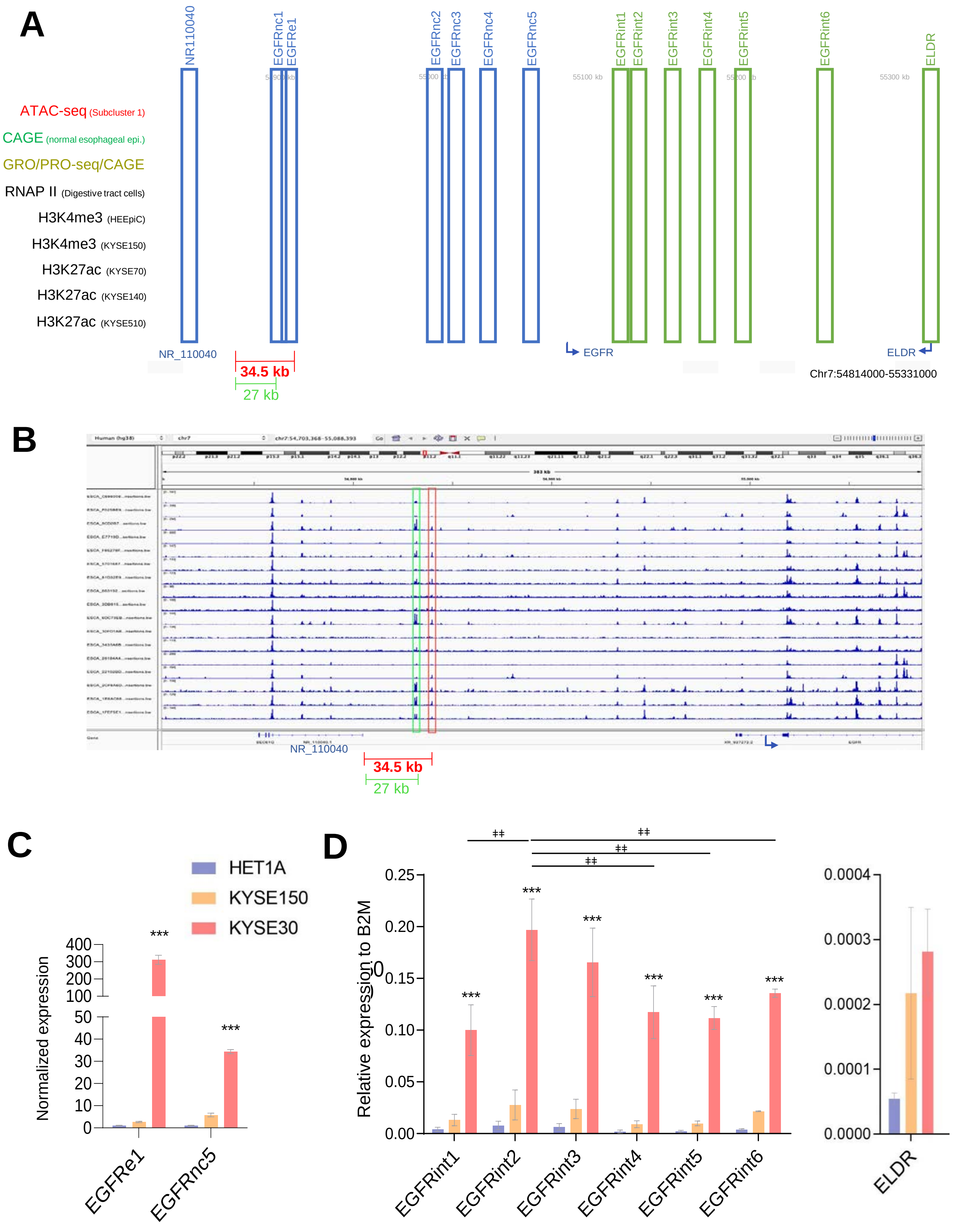

Choi, et al. Figure S3

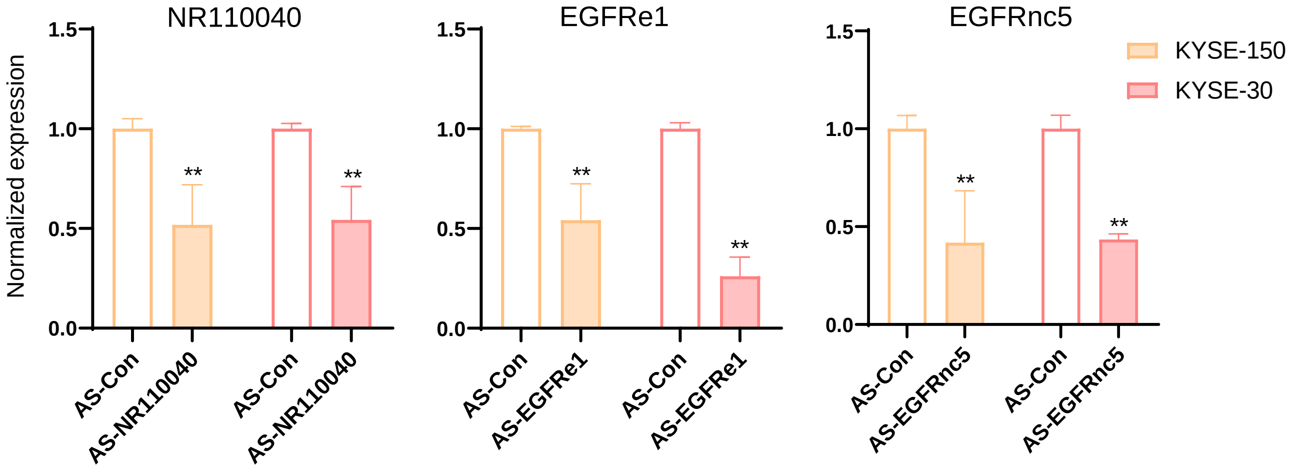

Choi, et al. Figure S4

Subcluster 3

Rank	Motif	P-value	% of Targets	% of Bgd	Known TF
1	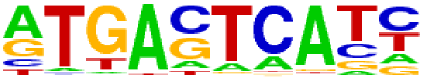	1e-183	18.64	2.64	FOSL1, BATF, ATF3, FOSL2, JUN, JUNB, JUND, FOS, FOSL2
2	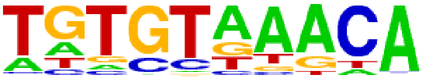	1e-19	18.43	11.22	FOXA2, FOX, FOXA1, FOXO3, FOXD1, FOXP1, FOXP2
3	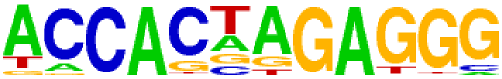	1e-18	1.41	0.13	CTCF, BORIS, RUNX2, ZNF354C, MYCN, USF2, RUNX

Subcluster 2

Rank	Motif	P-value	% of Targets	% of Bgd	Known TF
1	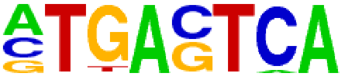	1e-18	13.17	3.33	BATF, ATF3, JUN, FOSL2, FOSL1, FOS, JUNB
2	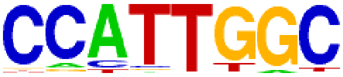	1e-12	13.62	4.66	NFY, NFIC, SOX3, NFYB
3	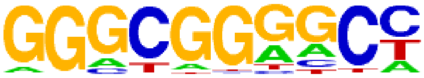	1e-10	28.79	16.33	SP1, KLF5, KLF4

Subcluster 4

Rank	Motif	P-value	% of Targets	% of Bgd	Known TF
1	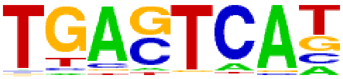	1e-29	16.53	2.52	BATF, FOSL2, FOSL1, ATF3, JUN, FOS, JUNB
2	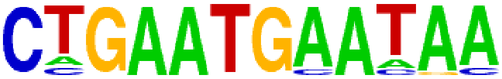	1e-12	1.38	0.00	PIT1, HNF1, BATF, JUN
3	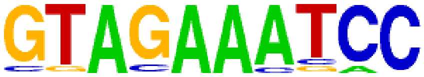	1e-11	3.03	0.12	PITX1, RELA, REL

Choi, et al. Figure S5

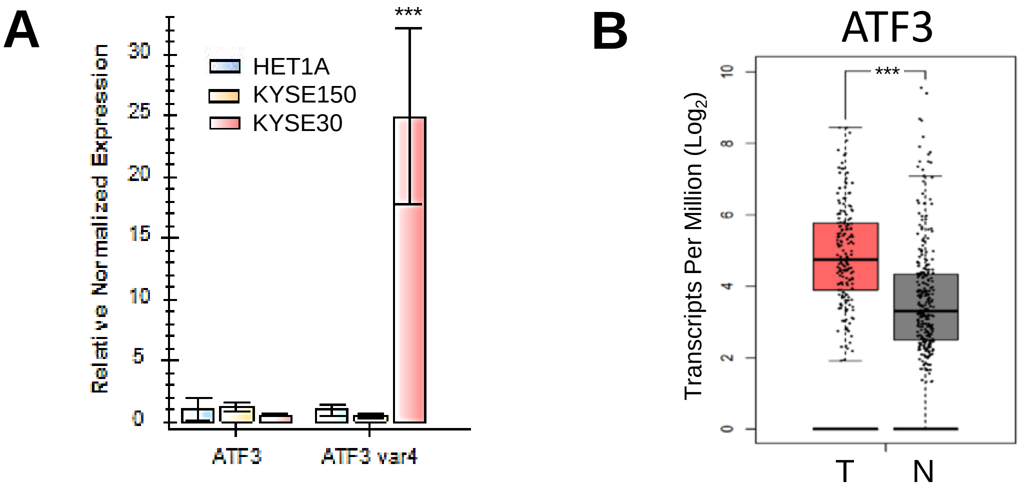

Choi, et al. Figure S6

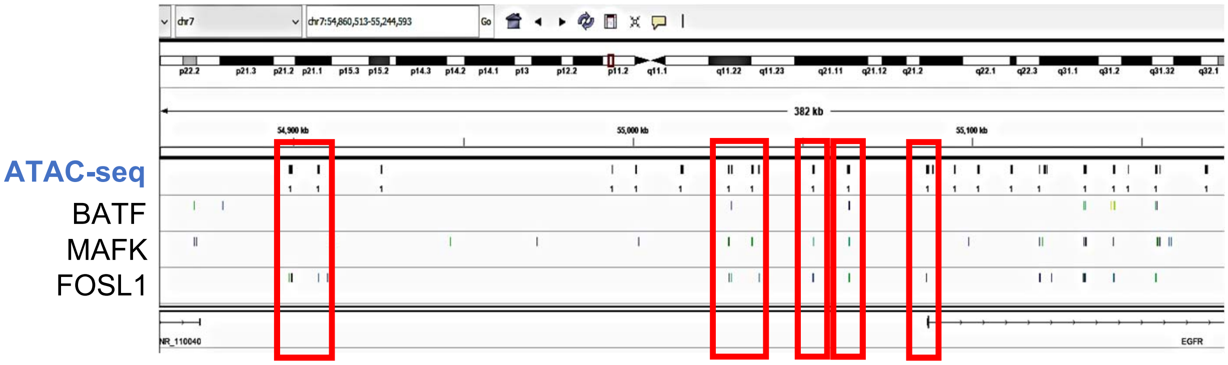

Choi, et al. Figure S7
